# Non-Monotonic Effect of Substrate Inhibition in Conjunction with Diffusion Limitation on the Response of Amperometric Biosensors

**DOI:** 10.3390/bios15070441

**Published:** 2025-07-09

**Authors:** Romas Baronas

**Affiliations:** Faculty of Mathematics and Informatics, Institute of Computer Science, Vilnius University, Didlaukio 47, LT-08303 Vilnius, Lithuania; romas.baronas@mif.vu.lt

**Keywords:** amperometric biosensor, substrate inhibition, diffusion limitation, transient response, mathematical modeling, computational simulation

## Abstract

The non-monotonic behavior of amperometric enzyme-based biosensors under uncompetitive and noncompetitive (mixed) substrate inhibition is investigated computationally using a two-compartment model consisting of an enzyme layer and an outer diffusion layer. The model is based on a system of reaction–diffusion equations that includes a nonlinear term associated with non-Michaelis–Menten kinetics of the enzymatic reaction and accounts for the partitioning between layers. In addition to the known effect of substrate inhibition, where the maximum biosensor current differs from the steady-state output, it has been determined that external diffusion limitations can also cause the appearance of a local minimum in the current. At substrate concentrations greater than both the Michaelis–Menten constant and the uncompetitive substrate inhibition constant, and in the presence of external diffusion limitation, the transient response of the biosensor, after immersion in the substrate solution, may follow a five-phase pattern depending on the model parameter values: it starts from zero, reaches a global or local maximum, decreases to a local minimum, increases again, and finally decreases to a steady intermediate value. The biosensor performance is analyzed numerically using the finite difference method.

## 1. Introduction

Enzyme-based amperometric biosensors were the first type of biosensors developed and remain the most popular due to their simplicity, ease of production, and low cost [1,2,3]. They measure changes in the output current at the working electrode caused by the direct oxidation or reduction of biochemical reaction products. The amperometric response is typically proportional to the analyte (substrate) concentration in a buffer solution [2,4,5,6]. These devices have found widespread applications in clinical, environmental, industrial, toxin detection, and other fields [3,7,8,9,10,11].

Most biosensors operate according to the Michaelis–Menten kinetics scheme,

(1) $$E+S\overset{k_{1}}{\underset{k_{-1}}{⇌}}\mathrm{ES}\overset{k_{2}}{\to }E+P,$$

where E is an enzyme, S is a substrate, ES is an enzyme–substrate complex, P is a reaction product, and $k_{1}$, $k_{-1}$ and $k_{2}$ are the rate constants [2,4,5,7].

Often, the kinetics of the enzyme-based biosensors are much more complicated than in the simplest scheme (1). Different substances may act as inhibitors and cause a reduction in the rate of an enzyme-catalyzed reaction [6,8,12,13]. The substrate in many enzyme-catalyzed reactions behaves as an inhibitor. In addition to the scheme (1), the interaction of the enzyme–substrate complex (ES) with other substrate molecules (S), resulting in the formation of a non-active inhibitory complex (ESS), can give rise to one of the simplest non-Michaelis–Menten mechanisms of enzyme action—uncompetitive substrate inhibition:

(2) $$\mathrm{ES}+S\overset{k_{3}}{\underset{k_{-3}}{⇌}}\mathrm{ESS},$$

where $k_{3}$ and $k_{-3}$ are the rate constants [2,3,6,8,14].

A second substrate molecule can also bind to a regulatory site on the free enzyme (E), leading to the formation of an inactive or less active complex (ESI). This phenomenon is referred to as competitive substrate inhibition:

(3) $$E+S\overset{k_{4}}{\underset{k_{-4}}{⇌}}\mathrm{ESI},$$

where $k_{4}$ and $k_{-4}$ are the rate constants [2,3,6,8,15,16].

In more complex systems involving noncompetitive (mixed) substrate inhibition, excess substrate can bind to both the free enzyme (E) and the enzyme–substrate complex (ES), resulting in the formation of inactive complexes in both pathways [12,14,17,18].

Understanding the kinetic properties of biosensors is crucial for their design and optimization [19,20,21]. Mathematical modeling has proven to be a useful tool to study the effect of enzyme inhibition [10,11,15,17,22,23,24,25]. Various approaches have been applied for the biosensor modeling [26,27,28,29,30]. Actual biosensors with substrate inhibition have already been modeled at various, often steady-state, conditions [16,18,31,32,33,34]. The amperometric biosensors utilizing the enzyme with the substrate inhibition have also been modeled at the external diffusion limitation and the steady-state [10,22] as well as the transition conditions [30,35,36].

In particular, Kulys showed that a multi-steady-state response can be generated at the electrode surface under external diffusion limitations when the substrate concentration is much greater than the Michaelis–Menten constant, assuming an extremely thin enzyme layer [22]. However, to the best of our knowledge, only non-monotonic transient responses featuring a maximum followed by a final steady-state current have been simulated [24,31,35,36,37,38]. In such cases, the response typically follows a three-phase pattern: starting from zero, reaching a maximum, and finally decreasing to a steady value.

When modeling practical biosensors, multi-layer models are usually required to achieve sufficient accuracy of the model [31,39,40]. Nevertheless, even mono-layer models that neglect external mass transport by diffusion have still been used in various applications in recent years due to the model’s simplicity [14,15,24,38,41,42,43,44,45]. However, external mass transport by diffusion significantly influences the dynamics of the catalytic processes in enzyme-loaded systems in general and biosensor response and sensitivity in particular [9,46,47,48,49,50]. Fortunately, mass transport through several outer diffusion layers can be rather effectively approximated by a single diffusion layer with effective diffusion coefficients [51,52,53]. As a result, two-compartment models have been widely used in biosensor modeling [10,39,54,55,56,57,58,59].

The aim of this work was to investigate in detail the influence of substrate inhibition, in conjunction with internal and external diffusion limitations, on the transient response of enzyme-based amperometric biosensors under uncompetitive, competitive, and noncompetitive (mixed) substrate inhibition. This study focuses on the conditions under which the transient response of the biosensor, after being immersed in a substrate solution, exhibits a complex, multi-phase pattern, characterized by the appearance of a local minimum, a local maximum, or even both.

Under transient conditions, a biosensor is mathematically modeled using a two-compartment model comprising a mono-enzyme layer, where both the enzymatic reaction and mass transport by diffusion occur, and a diffusion-limiting region, where only mass transport by diffusion takes place. The model is based on a system of reaction–diffusion equations that includes a nonlinear term associated with non-Michaelis–Menten kinetics of the enzymatic reaction and accounts for partitioning between layers. The performance of the treated system is analyzed numerically using the finite difference technique [60,61], and the simulation results are compared with previous studies on biosensors under substrate inhibition [22,24,31,35,36].

## 2. Mathematical and Computational Modeling

### 2.1. Biosensor Principal Structure

**Figure 1:**
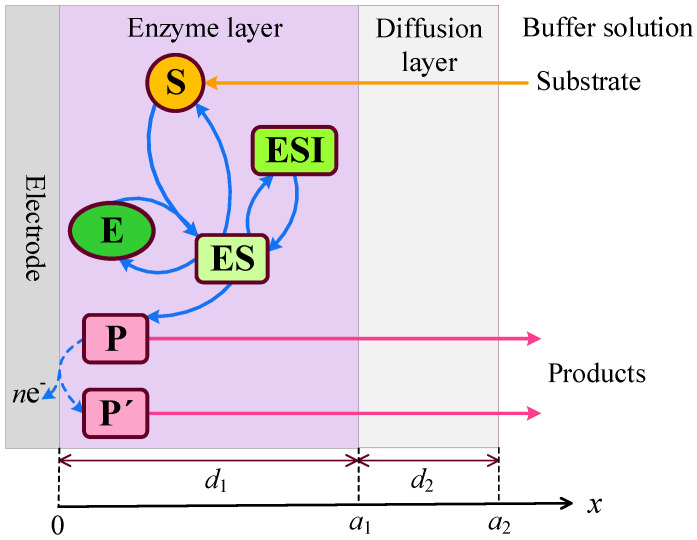
Schematic representation of the amperometric biosensor. The figure is not to scale.

An amperometric biosensor consists of an electrode coated with a relatively thin enzyme layer, also referred to as the enzyme membrane [2,4,6,7,9]. The model describes three distinct regions: the enzyme layer, where both enzymatic reactions and diffusion-driven mass transport occur; a diffusion-limiting region, where only diffusion-based mass transport takes place; and a convective region, where the analyte concentration remains constant [35,36]. A schematic representation of the biosensor model is shown in Figure 1, where $d_{1}$ and $d_{2}$ denote the thicknesses of the enzyme layer and the outer diffusion layer, respectively.

In the enzyme layer, we account for the enzyme-catalyzed reactions described by Equations (1)–(3). At the electrode surface, the electro-active product P is converted into a non-interfering species $P^{\prime }$, releasing electrons in the process,

(4) $$P\to P^{\prime }\pm ne^{-}$$

where *n* is the number of electrons transferred in the reaction.

Some reactions in the network (1)–(4) occur very rapidly, while others proceed much more slowly [2,4,6,7,9]. This wide disparity in reaction timescales complicates both the simulation of the network dynamics and the understanding of its fundamental behavior. To address these challenges, the quasi-steady-state approximation (QSSA) is commonly employed [62,63].

Under the assumption of the QSSA, the concentrations of the enzyme (E) and the intermediate complexes (ES, ESS, and ESI) remain constant over time. As a result, the rate of the biochemical reaction is described by the following equation, which does not follow Michaelis–Menten kinetics:

(5) $$\begin{array}{cc} \; & V\left(S\right)=\frac{V_{\max}S}{K_{M}\left(1+S/K_{I}^{\prime }\right)+S\left(1+S/K_{I}\right)}\;,\\ V_{\max}=k_{2}E_{0}, & \;K_{M}=\left(k_{-1}+k_{2}\right)/k_{1},\;K_{I}=k_{-3}/k_{3},\;K_{I}^{\prime }=k_{-4}/k_{4}, \end{array}$$

where *S* is the substrate concentration, $V_{\max}$ is the maximal enzymatic rate, $E_{0}$ is the total enzyme concentration, $K_{M}$ is the Michaelis constant, and $K_{I}$ and $K_{I}^{\prime }$ are the inhibition (dissociation) constants [4,14,16,17,64,65].

At very low substrate concentrations, where $S\ll K_{M}$, $S\ll K_{I}$ and $S\ll K_{I}^{\prime }$, the nonlinear reaction rate given in (5) simplifies to the first-order reaction rate $V_{\max}S/K_{M}$. At high substrate concentrations such that $S\gg K_{M}$ but still $S\ll K_{I}$ and $S\ll K_{I}^{\prime }$, the rate becomes independent of the substrate concentration, exhibiting zero-order kinetics. The influence of inhibition on the overall biochemical process decreases as the inhibition constants $K_{I}$ and $K_{I}^{\prime }$ increase, and the kinetics gradually approach the standard Michaelis–Menten kinetics form as $K_{I}\to \infty$ and $K_{I}^{\prime }\to \infty$.

In the case of uncompetitive substrate inhibition ($K_{I}^{\prime }\to \infty$), where only two enzymatic reactions (1) and (2) occur, the reaction rate reduces to:

(6) $$V\left(S\right)=\frac{V_{\max}S}{K_{M}+S+S^{2}/K_{I}}\;,\;K_{I}^{\prime }\to \infty \;.$$

When $S>K_{I}$, the inhibitory term $S^{2}/K_{I}$ in the denominator becomes significant, and the reaction rate begins to decline despite the increasing substrate concentration [4,64].

When the biosensor operates under competitive substrate inhibition (with $K_{I}\to \infty$), the reaction rate can be rewritten in Michaelis–Menten form,

(7) $$\begin{array}{cc} \; & V\left(S\right)=\frac{V_{\max}S}{K_{M}\left(1+S/K_{I}^{\prime }\right)+S}=\frac{V_{\max}^{\mathrm{eff}}S}{K_{M}^{\mathrm{eff}}+S}\;,\;K_{I}\to \infty ,\\ \; & V_{\max}^{\mathrm{eff}}=\frac{V_{\max}}{1+K_{M}/K_{I}^{\prime }}\;,\;K_{M}^{\mathrm{eff}}=\frac{K_{M}}{1+K_{M}/K_{I}^{\prime }}\;. \end{array}$$

Here, $V_{\max}^{\mathrm{eff}}$ and $K_{M}^{\mathrm{eff}}$ represent the apparent or effective kinetic parameters under competitive substrate inhibition. They correspond to the effective maximal enzymatic rate and Michaelis constant, respectively. Thus, the behavior of enzyme-based biosensors under competitive substrate inhibition can be described using modified kinetic expressions that extend beyond the classical Michaelis–Menten model.

The biochemical reactions occurring in practical biosensors are influenced by various important biosensor-related factors such as pH, enzyme concentration, cofactor availability, temperature, enzyme degradation over time, and others [2,4,5,7,12,15,25]. Under certain conditions, these factors are commonly incorporated into the Michaelis–Menten parameters and inhibition constants. Although they are not part of the original Michaelis–Menten derivation, they are often embedded empirically through experimental observations. In biosensor modeling, these adjusted parameters are typically referred to as the “apparent” or “effective” $V_{\max}^{\mathrm{eff}}$ and $K_{M}^{\mathrm{eff}}$ [8,9,12,14,30,58].

This work focuses on the impact of uncompetitive and noncompetitive substrate inhibition and is restricted to substrate concentrations greater than at least one of the inhibition constants, $K_{I}$ or $K_{I}^{\prime }$.

In the two-compartment (or two-layer) model, the diffusion layer is commonly considered to be the Nernst diffusion layer [9,60,66]. However, if the external Nernst diffusion layer is neglected, the diffusion layer can instead be modeled as a semi-permeable (diffusion-limiting) membrane [30,39,48,60,67]. It is important to note, though, that a Nernst layer with zero thickness cannot be realized in practice [68].

Mathematical models of biosensors sometimes account for both the outer membrane and the Nernst diffusion layer [31,52,53,69]. However, the mass transport across multiple diffusion layers can often be effectively approximated by a single layer with adjusted diffusion coefficients, allowing a complex multi-compartment model to be simplified into a two-compartment one [51,52,53]. As a result, the effects examined in this study are also applicable to amperometric biosensors modeled with several diffusion layers, including both the outer membrane and the Nernst layer.

### 2.2. Mathematical Model

Assuming symmetrical geometry of the electrode, enzymatic, and diffusion layers, as well as uniform distribution of the immobilized enzyme within the enzyme membrane, leads to a two-compartment mathematical model defined in a one-dimensional spatial domain. This model is expressed as an initial boundary value problem that captures the dynamics of substrate and product concentrations [8,30,39,70].

#### 2.2.1. Governing Equations

The changes in the concentrations of the substrate S and product P within the enzyme layer over time are governed by a system of reaction–diffusion equations (t>0),

(8) $$\begin{array}{cc} \frac{\partial S_{1}}{\partial t} & =D_{S_{1}}\frac{\partial^{2}S_{1}}{\partial x^{2}}-\frac{V_{\max}S_{1}}{K_{M}\left(1+S_{1}/K_{I}^{\prime }\right)+S_{1}\left(1+S_{1}/K_{I}\right)}\;,\\ \frac{\partial P_{1}}{\partial t} & =D_{P_{1}}\frac{\partial^{2}P_{1}}{\partial x^{2}}+\frac{V_{\max}S_{1}}{K_{M}\left(1+S_{1}/K_{I}^{\prime }\right)+S_{1}\left(1+S_{1}/K_{I}\right)}\;,\;x\in \left(0,\;a_{1}\right), \end{array}$$

where $S_{1}\left(x,t\right)$ and $P_{1}\left(x,t\right)$ are the concentrations of the substrate and the product in the enzyme layer, $D_{S_{1}}$ and $D_{P_{1}}$ are the diffusion coefficients, $d_{1}=a_{1}$ is the thickness of the enzyme layer, and $V(S_{1})$ is the reaction rate, as defined in (5) [8,39,64].

In the diffusion layer, both compounds are transported solely by diffusion (t>0),

(9) $$\begin{array}{cc} \frac{\partial S_{2}}{\partial t} & =D_{S_{2}}\frac{\partial^{2}S_{2}}{\partial x^{2}},\\ \frac{\partial P_{2}}{\partial t} & =D_{P_{2}}\frac{\partial^{2}P_{2}}{\partial x^{2}},\;x\in \left(a_{1},\;a_{2}\right), \end{array}$$

where $S_{2}\left(x,t\right)$ and $P_{2}\left(x,t\right)$ are the concentrations of the substrate and the reaction product, $D_{S_{2}}$, $D_{P_{2}}$ are the diffusion coefficients, $a_{2}=a_{1}+d_{2}$, and $d_{2}$ is the thickness of the diffusion layer [8,30,39].

#### 2.2.2. Boundary Conditions

During biosensor operation, the concentrations of the substrate and product remain constant in the bulk solution (t>0),

(10) $$S_{2}\left(a_{2},t\right)=S_{0},\;P_{2}\left(a_{2},\;t\right)=0,$$

where $S_{0}$ is the concentration of the substrate in the bulk.

At the boundary between adjacent layers, the fluxes of the substrate and product are assumed to be equal; that is, the outgoing flux from one layer equals the incoming flux to the next. However, the concentrations on either side of the interface differ and are related by the formal partition coefficients $\theta_{S}$ for the substrate and $\theta_{P}$ for the product (t>0) [69,70,71,72,73],

(11) $$\begin{array}{cc} \; & D_{S_{1}}\frac{\partial S_{1}}{\partial x}{|}_{x=a_{1}}=D_{S_{2}}\frac{\partial S_{2}}{\partial x}{|}_{x=a_{1}},\;S_{1}\left(a_{1},t\right)=\theta_{S}S_{2}\left(a_{1},t\right),\\ \; & D_{P_{1}}\frac{\partial P_{1}}{\partial x}{|}_{x=a_{1}}=D_{P_{2}}\frac{\partial P_{2}}{\partial x}{|}_{x=a_{1}},\;P_{1}\left(a_{1},t\right)=\theta_{P}P_{2}\left(a_{1},t\right). \end{array}$$

The partition coefficients are generally different for different species [72,74], but they are often assumed to be identical [10,53,58,69,71].

Due to the electrochemical reaction (4), the concentration of the reaction product decreases at the electrode surface. The substrate is considered electrically inactive, and no concentration flux is assumed for it (t>0) [8,39,75],

(12) $$P_{1}\left(0,t\right)=0,\;D_{S_{1}}\frac{\partial S_{1}}{\partial x}{|}_{x=0}=0,\;t>0.$$

#### 2.2.3. Initial Conditions

Two different initial conditions corresponding to two modes of biosensor operation are considered.

In the first mode, the biosensor is assumed to be permanently immersed in a buffer solution, and its operation begins when the analyte (substrate) is introduced at t=0 into the buffer solution,

(13) $$\begin{array}{cc} \; & S_{1}\left(x,0\right)=0,\;\;P_{1}\left(x,0\right)=0,\;x\in \left[0,\;a_{1}\right],\\ \; & S_{2}\left(x,0\right)=0,\;\;P_{2}\left(x,0\right)=0,\;x\in \left[a_{1},a_{2}\right),\\ \; & S_{2}\left(a_{2},\;0\right)=S_{0},\;P_{2}\left(a_{2},\;0\right)=0. \end{array}$$

This setup simulates injection analysis (IA) or real-time monitoring, where the analyte arrival initiates the biosensor transient response [8,9,10,15,31,56,76].

The second type of operation is common in batch analysis (BA), when the biosensor is directly immersed in a buffer solution containing the analyte [8,9,25,58,77]. The biosensor operation starts responding to the analyte from the moment of immersion. In this case, the initial conditions (13) have to be replaced with the following:

(14) $$\begin{array}{cc} \; & S_{1}\left(x,0\right)=0,\;\;P_{1}\left(x,0\right)=0,\;x\in \left[0,\;a_{1}\right),\\ \; & S_{1}\left(a_{1},\;0\right)=\theta_{S}S_{0},\;P_{1}\left(a_{1},\;0\right)=0,\\ \; & S_{2}\left(x,0\right)=S_{0},\;P_{2}\left(x,0\right)=0,\;x\in \left[a_{1},a_{2}\right]. \end{array}$$

Let us notice that both initial conditions, (13) and (14), result in the same steady-state solution for the problem (8)–(12). Only the transient solution is affected by the initial conditions [8,30,39,60].

The two-compartment model defined by Equations (8)–(14) converges to the corresponding one-compartment model as $a_{2}\to a_{1}$ ($d_{2}\to 0$) [15,38,41,42,44,45].

### 2.3. Biosensor Response

The amperometric electrode detects the faradaic current, whether anodic or cathodic in nature [2,8,64]. The current density I(t) at time *t* can be explicitly derived using Faraday’s and Fick’s laws,

(15) $$I\left(t\right)=nFD_{P_{1}}\frac{\partial P_{1}}{\partial x}{|}_{x=0},$$

where *n* is the number of electrons involved in a charge transfer at the electrode surface, and *F* is the Faraday constant [8,39,75].

As t→∞, the system (8)–(12) approaches a steady state [8,39,64],

(16) $$I_{\mathrm{ss}}=\lim_{t\to \infty }I\left(t\right),$$

where $I_{\mathrm{ss}}$ is the density of the steady-state output current.

Since the transient current in the case of the enzyme inhibition can be a non-monotonic function of time, the maximal current has also been used as a characteristic for this kind of biosensor [24,31,35,36,37,38]. Aiming to determine conditions under which biosensor response follows a multi-phase pattern, the number $N_{\mathrm{ext}}$ of extrema in transient output current I(t) was studied. A local or global maximum of output current I(t) occurs at time $t=t_{e}$ if $I\left(t_{e}\right)\geq I\left(t\right)$ for all *t* near $t_{e}$, while a local minimum occurs at that time $t=t_{e}$ if $I\left(t_{e}\right)\leq I\left(t\right)$ for all *t* near $t_{e}$. $N_{\mathrm{ext}}$ is considered as the total count of maxima and minima that the function I(t) has at t>0,

(17) $$N_{\mathrm{ext}}=|\left\{t>0\;|\;\frac{dI(t)}{dt}=0\;\mathrm{and}\;\frac{d^{2}I\left(t\right)}{dt^{2}}\neq 0\right\}|,$$

where |·| denotes the set cardinality and the condition for defining the set.

In the specific case of a monotonic output current, I(t), $N_{\mathrm{ext}}$ = 0. In the case of a three-phase pattern, when the output current starts from zero, reaches a maximum, and then decreases to a steady value, $N_{\mathrm{ext}}$ = 1. $N_{\mathrm{ext}}$ = 2 when the biosensor response approaches a four-phase pattern, exhibiting a local minimum in addition to the maximum.

### 2.4. Dimensionless Model Parameters

To identify the key governing parameters of the mathematical model, a dimensionless form is typically derived [66,69]. The two-compartment model (8)–(12) was transformed into a dimensionless form by rescaling time and space [30,36,48]. This process yielded the following dimensionless governing parameters:

(18) $$\begin{array}{cc} x^{*}=\frac{x}{a_{1}},\;t^{*} & =\frac{D_{S_{1}}t}{a_{1}^{2}},\;S_{0}^{*}=\frac{S_{0}}{K_{M}},\;K_{I}^{*}=\frac{K_{I}}{K_{M}},\;K_{I}^{\prime }*=\frac{K_{I}^{\prime }}{K_{M}},\\ S_{i}^{*} & =\frac{S_{i}}{K_{M}},\;P_{i}^{*}=\frac{P_{i}}{K_{M}},\;i=1,2,\\ \sigma^{2}=\frac{V_{\max}d_{1}^{2}}{K_{M}D_{S_{1}}} & ,\;\beta_{S}=\frac{D_{S_{2}}d_{1}}{\theta_{S}D_{S_{1}}d_{2}},\;\beta_{P}=\frac{D_{P_{2}}d_{1}}{\theta_{P}D_{P_{1}}d_{2}},\\ I^{*}\left(t^{*}\right) & =\frac{I\left(t\right)\;d_{1}}{n_{e}FD_{S_{1}}K_{M}},\;I_{ss}^{*}=\lim_{t^{*}\to \infty }I^{*}\left(t^{*}\right), \end{array}$$

where $\sigma^{2}$ is the dimensionless Damköhler number (the Thiele modulus squared) or the diffusion module, and $\beta_{S}$ and $\beta_{P}$ are the Biot numbers for the substrate and product, respectively [39,75,78,79]. Equations (8)–(12) in dimensionless form are presented in the Appendix A. All of the dimensional and dimensionless model parameters are listed in Table A1.

The dimensionless parameter $\sigma^{2}$ represents the ratio between the intrinsic enzyme reaction rate ($V_{\max}/K_{M}$) and the rate of substrate diffusion through the enzyme layer ($D_{S_{1}}/d_{1}^{2}$). When $\sigma^{2}\ll 1$, the biosensor response is governed primarily by enzyme kinetics. In contrast, when $\sigma^{2}\gg 1$, internal diffusion becomes the limiting factor in the response [2,8,39,47,79]. By rearranging the expression for $\sigma^{2}$, one can derive the characteristic timescales for both the enzymatic reaction and internal diffusion.

The Biot number is a dimensionless parameter commonly used to compare the relative resistances to mass transport caused by external and internal diffusion processes [9,46,47,49,50,78]. Because the diffusion characteristics of the substrate and product typically differ, separate Biot numbers are often defined for each. However, these are frequently assumed to be equal for simplicity [46,48,49,50,53,80]. A high Biot number suggests that diffusion within the enzyme layer is slower than in the surrounding diffusion layer, whereas a low Biot number indicates that diffusion is more restricted in the diffusion layer than in the enzyme layer [9,47,78].

### 2.5. Numerical Simulation

Due to the nonlinearity of the governing Equation (8), the initial boundary value problem (8)–(14) can be analytically solved only for specific values of the model parameters [10,39,60]. Hence, the problem was solved numerically.

To find a numerical solution to the problem (8)–(14), a non-uniform discrete grid was introduced in space and time. A semi-implicit linear finite difference scheme has been built as a result of the difference approximation of the model equations [30,36,81]. The resulting system of linear algebraic equations was solved efficiently using the Thomas (the tridiagonal matrix) algorithm [60]. To have an accurate and stable result, it was required to use a small step size in the *x* direction at the boundaries x=0, $x=a_{1}$, and $x=a_{2}$, where the concentration gradients are larger than the gradients away from those boundaries. Further from these boundaries, an exponentially increasing step size was used [30,53,80].

Although the time step is restricted by the partition conditions (11) [82,83,84], it was reasonable to apply an increasing step size in the time direction [85], as the biosensor action follows the steady-state assumption as t→∞. The final step size in time was a few orders of magnitude higher than the first one [48]. The density $I_{\mathrm{ss}}$ of the steady-state output current was approximated by the output current calculated at the moment, when the normalized absolute current slope value fell below a given small value 0.001 [30,39].

The numerical simulator has been programmed in Java [86]. The numerical solution was validated using exact analytical solutions known for specific cases of the first and zero-order reaction rates at the steady-state conditions [39,48] and numerical solutions derived for a two-compartment model of amperometric biosensors at transient conditions [10,36,39]. Approximate analytical solutions, obtained for the corresponding one-compartment model of biosensors with substrate inhibition at steady-state [33,41,42] and transient conditions [38], were also used for validation of the numerical solution.

The simulation results were visualized using Origin [87].

## 3. Results and Discussion

To investigate the non-monotonic behavior of amperometric enzyme-based biosensors under substrate inhibition, in conjunction with internal and external diffusion limitations, the biosensor action was simulated across a wide range of key dimensionless model parameter values, using the following typical assumptions for the parameter values [1,35,36,48,53]:

(19) $$\begin{array}{c} D_{P_{1}}=D_{S_{1}}=400\;\mu m^{2}/s,\;D_{P_{2}}=D_{S_{2}}=600\;\mu m^{2}/s,\\ \theta_{P}=\theta_{S}=\theta ,\;\beta_{P}=\beta_{S}=\beta ,\\ d_{1}=20\;\mu m,\;d_{2}=300\;\mu m,\;K_{M}=100\;\mu M,\;n_{e}=1. \end{array}$$

The biosensor response behavior is primarily analyzed under uncompetitive substrate inhibition ($K_{I}^{\prime }=\infty$). Additionally, the response is examined in the case of mixed (noncompetitive) substrate inhibition.

### 3.1. Temporal Dynamics of Biosensor Response

**Figure 2:**
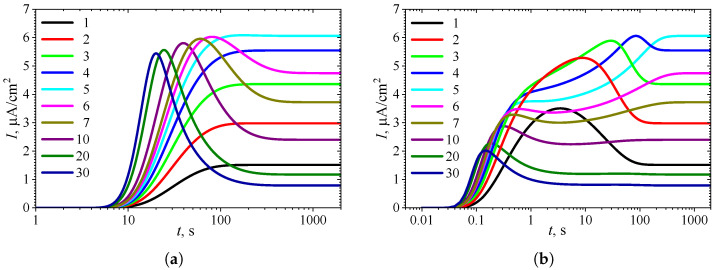
Dynamics of the output current I(t) at ten values of the normalized substrate concentration $S_{0}^{*}$, with fixed parameters $V_{\max}$ = 100μM/s, θ = 0.75, $K_{I}=K_{M}$ and $K_{I}^{\prime }=\infty$, in IA (**a**) and BA (**b**) modes. Other parameters are defined in (19).

Figure 2 shows the typical temporal dynamics of the biosensor current *I*, simulated under uncompetitive substrate inhibition ($K_{I}^{\prime }=\infty$) at the following ten initial substrate concentrations $S_{0}$: 0.1, 0.2, 0.3, 0.4, 0.5, 0.6, 0.7, 1, 2, and 3 mM. The simulations were performed using fixed parameters: $V_{\max}$ = 100μM/s, θ = 0.75, and $K_{I}=K_{M}$, in both types of analysis, injection analysis (IA) and batch analysis (BA). The corresponding normalized values $S_{0}^{*}=S_{0}/K_{M}$ of the substrate concentration are indicated on the curves in Figure 2. All the other model parameters were defined in (19). These simulations were performed under mixed control, involving both enzyme kinetics and internal diffusion ($\sigma^{2}=1$). On the other hand, mass transport in the outer diffusion layer was slower than in the enzyme layer, as indicated by β=0.13.

One can see a noticeable difference in the dynamics of the biosensor response in Figure 2 when changing the substrate concentration $S_{0}$. The shape of the curves also depends significantly on the mode of analysis, i.e., on the initial conditions (13) or (14). However, the steady-state response is independent of the analysis mode, as the steady-state solution of the initial boundary value problem (8)–(11) is unaffected by those initial conditions [8,30,39,60].

At the beginning of biosensor operation, the output current becomes noticeably slower in IA mode than in BA mode. The delay consists of about 6–10 s. This delay in the transient response can be attributed to the diffusion time required for the substrate to pass through the outer diffusion layer and reach the enzyme layer in IA mode [4,34,64]. In contrast, in BA, the substrate contacts the enzyme layer immediately at t=0. The corresponding steady-state times are approximately the same, though they noticeably depend on the substrate concentration.

In IA (Figure 2a), at relatively high substrate concentrations ($S_{0}^{*}>5$), the response follows a three-phase pattern: starting from zero, reaching a maximum, and finally decreasing to a steady value. The output current, after reaching its maximum, enters the descending limb of the bell-shaped curve characteristic of uncompetitive substrate inhibition. In the case of low and moderate substrate concentrations ($S_{0}^{*}\leq 5$ in Figure 2a), the biosensor current monotonically approaches steady-state. On the other hand, Figure 2a shows nonmonotonic behavior of the steady-state current. At substrate concentrations corresponding to the three-phase pattern ($S_{0}^{*}>5$), the steady-state current decreases with increasing $S_{0}^{*}$, whereas at lower concentrations ($S_{0}^{*}\leq 5$), it increases with increasing $S_{0}^{*}$. These aspects of the biosensor with uncompetitive substrate inhibition are well known [24,31,35,36,37,38].

In particular, Forastiere et al. determined that the maximal steady-state current can be achieved at relatively low substrate concentration due to substrate inhibition, and that it can be several times greater than the current observed at extremely high substrate concentrations [24], consistent with the simulation results presented in Figure 2. Rafat et al. have demonstrated a nonmonotonic dependence of the biosensor response and sensitivity on the substrate concentration and mass transfer for IBE biosensors and an amperometric electrochemical immunosensor [10,58]. Sánchez-Trasviña et al. demonstrated how the appearance of substrate inhibition transforms the monotonically increasing reaction rate versus luminol concentration into a nonmonotonic behavior [65].

Figure 2b shows noticeably more complex dynamics of the biosensor current in BA mode than in IA. Even at relatively low substrate concentrations ($1\leq S_{0}^{*}\leq 4$), the output current is nonmonotonic. At a slightly greater concentration $S_{0}^{*}=5$, the output current becomes a monotonously increasing function of time *t*, butit has an extra inflection point (t≈2 s) where the curve changes from concave down to concave up.

In the case of relatively high substrate concentrations ($6\leq S_{0}^{*}\leq 10$), the biosensor response exhibits a local minimum and follows a four-phase pattern. At higher concentrations $S_{0}^{*}\geq 20$, the response even approaches a five-phase pattern, although the oscillations at t>10 are only slight. Specifically, at $S_{0}^{*}=20$, the transient current I(t) starts from zero, reaches a global maximum of $2.27\;\mu A/cm^{2}$ at t=0.18 s, decreases to a local minimum of $1.196\;\mu A/cm^{2}$ at t=10.5 s, increases to a local maximum of $1.206\;\mu A/cm^{2}$ at t=42 s, and finally decreases to a steady value of $1.17\;\mu A/cm^{2}$. Thus, the variation between the local extrema and the steady value is only 2–3%. At a higher concentration of $S_{0}^{*}=30$, this variation is even smaller.

### 3.2. Effect of Internal Diffusion Limitation

**Figure 3:**
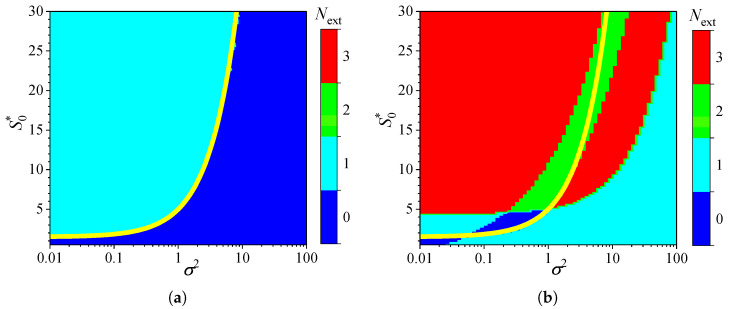
Number $N_{\mathrm{ext}}$ of extrema of the output current vs. the diffusion module $\sigma^{2}$ and the normalized substrate concentration $S_{0}^{*}$, with fixed parameters θ = 0.75, $K_{I}=K_{M}$ and $K_{I}^{\prime }=\infty$, for IA (**a**) mode and BA (**b**) mode. Other parameters are defined in (19). The yellow line is defined in (20).

Figure 2 shows the influence of substrate concentration on the dynamics of output current at a fixed maximal enzymatic rate of $V_{\max}$ = 100μM/s, which corresponds to a diffusion module equal to unity ($\sigma^{2}=1$). To investigate the effect of the diffusion module on the transient response of the amperometric biosensors, the response was simulated at very different values of $V_{\max}$. This allowed the transition from enzyme kinetics control ($\sigma^{2}\ll 1$) to internal diffusion control ($\sigma^{2}\gg 1$) to be observed, while keeping all other parameters the same as those used in the simulations depicted in Figure 2. Figure 3 shows the number $N_{\mathrm{ext}}$ of extrema calculated from the simulated responses in both modes of analysis, IA and BA.

As shown in Figure 3a, the transient output current exhibits one or even no local extrema in IA, when the diffusion module changes in four orders of magnitude, from 0.01 to 100, and the dimensionless substrate concentration $S_{0}^{*}$ changes from 1 to 30. When the biosensor acts under the internal diffusion limitation ($\sigma^{2}\gg 1$), the response follows a two-phase pattern ($N_{\mathrm{ext}}=0$): starting from zero increases to a steady value. A three-phase pattern ($N_{\mathrm{ext}}=1$) is observed when the biosensor response is governed by enzyme kinetics or mixed control ($\sigma^{2}≲10$), although it also depends on the substrate concentration. The yellow line in Figure 3 represents an approximate boundary between the two values of $N_{\mathrm{ext}}$, 0 and 1, i.e., between model parameter values that result in either two-phase or three-phase patterns. This yellow line is a linear approximation of the boundary,

(20) $$S_{0}^{*}=1.5+3.5\sigma^{2}.$$

The relationship (20) between the substrate concentration and the diffusion module $\sigma^{2}$, resulting in changes in the number of response phases, is approximately linear when the biosensor operates in IA mode, as defined by (20) (Figure 3a). Similar dependencies of the substrate concentration on the diffusion module have already been observed [31,36,38,44,45]. In particular, it was found that the minimum dimensionless substrate concentration at which the response reaches its maximum is a monotonically increasing function of $\sigma^{2}$ [36]. In the case of BA (Figure 3b), that relation is noticeably more complicated as the number $N_{\mathrm{ext}}$ of extrema varies between zero and three, indicating that the number of phases in the response pattern ranges between two and five. In particular, at $\sigma^{2}=1$, increasing the normalized substrate concentration $S_{0}^{*}$ from 1 to 30 results in the number $N_{\mathrm{ext}}$ of extrema changing in the following sequence: 1, 0, 1, 2, 3. This can also be noticed in Figure 2b.

Although the variation in the number of extrema $N_{\mathrm{ext}}$ differs noticeably among the analysis modes, at relatively low substrate concentrations ($S_{0}^{*}≲4.5$) and very low values of the diffusion module ($\sigma^{2}≲0.03$), the number $N_{\mathrm{ext}}$ is practically invariant across the analysis mode, as observed in the lower left corners of Figure 3a,b.

**Figure 4:**
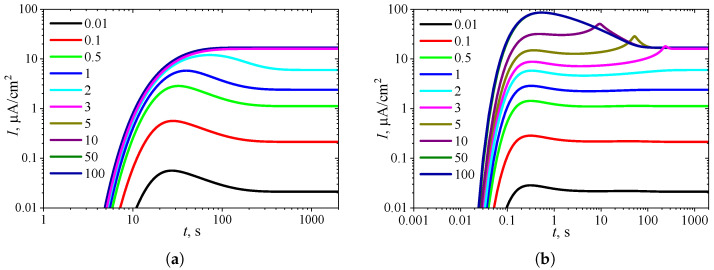
Dynamics of the output current I(t) at ten values of the diffusion module $\sigma^{2}$, with fixed substrate concentration $S_{0}^{*}$ = 10 and $K_{I}^{\prime }=\infty$, in IA (**a**) mode and BA (**b**) mode. Other parameters are the same as in Figure 3.

To observe the effect of the diffusion module $\sigma^{2}$ on the shape of the transient response, the biosensor action was simulated at different values of $\sigma^{2}$ while keeping the substrate concentration fixed at a relatively high level ($S_{0}^{*}$ = $10K_{I}^{*}$ = 10, $S_{0}=10K_{I}$), where the uncompetitive inhibition plays a significant role in the biosensor response. The simulation results are shown in Figure 4.

One can see in Figure 4a that in IA, the transient output current exhibits a global maximum for $\sigma^{2}<3$, whereas it is a monotonously increasing function of time *t* for greater values of the diffusion module $\sigma^{2}$, as predicted in Figure 3a. In BA (Figure 4b), for $\sigma^{2}<3$, the shape of I(t) is similar to that observed in the IA mode (Figure 4a), although the function I(t) in BA has additional local extrema, which are close to steady values. However, at slightly greater values of $\sigma^{2}$ ($3\leq \sigma^{2}\leq 10$), the transient current I(t) exhibits a noticeable peak in BA. At high values of the diffusion module ($\sigma^{2}>20$), when the response is governed by internal diffusion control, the output current exhibits only a global maximum, following a two-phase pattern.

On the other hand, at high values of the diffusion module ($\sigma^{2}>5$), the transient response in IA becomes practically invariant to $\sigma^{2}$, whereas in BA, the response dynamics still noticeably depend on $\sigma^{2}$. Maintaining the analytical capability of biosensors for as long as possible is very important [2,4,7]. Typically, the maximal enzymatic rate $V_{\max}$ decreases over time due to enzyme inactivation [76,88]. Therefore, ensuring the stability of the biosensor response (the biosensor resistance) across a range of $V_{\max}$ values is crucial [30,38,42,44,45,88]. Since $V_{\max}$ directly influences $\sigma^{2}$, it is essential to maintain a stable response even when $\sigma^{2}$ undergoes slight variations.

In particular, at two significantly different values of $\sigma^{2}$, namely 0.5 and 5, the response of a biosensor operating under BA conditions follows a five-phase pattern ($N_{\mathrm{ext}}$ = 3). However, in the case of $\sigma^{2}=0.5$ (and for $\sigma^{2}<0.5$ as well), the local minimum is barely noticeable, whereas for $\sigma^{2}=5$, all extrema and all five phases are clearly observable. This is particularly important in practical applications of amperometric biosensors, as oscillations in the biosensor response may complicate the use of the calibration curve [21,22]. On the other hand, analyzing both the steady-state and the maximal biosensor currents can significantly extend the calibration curve when using intelligent biosensors [21,35,36,89].

### 3.3. Effect of External Diffusion Limitation

**Figure 5:**
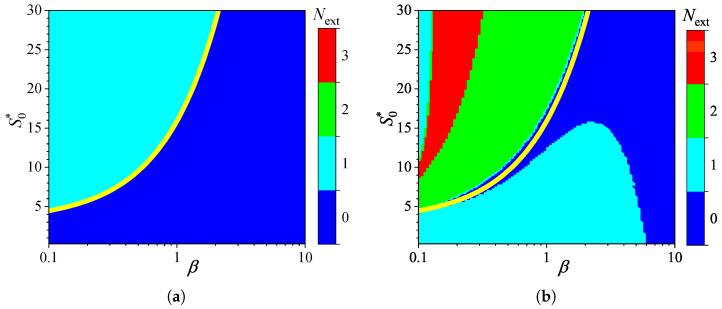
Number $N_{\mathrm{ext}}$ of extrema of the biosensor current vs. the Biot number β and the normalized substrate concentration $S_{0}^{*}$, with fixed parameters $V_{\max}$ = 100μM/s, $K_{I}=K_{M}$ and $K_{I}^{\prime }=\infty$, for IA (**a**) mode and BA (**b**) mode. Other parameters are defined in (19). The yellow line is defined in (21).

To investigate the influence of external diffusion limitations on the behavior of amperometric enzyme-based biosensors, the biosensor response was simulated by varying the partition coefficient θ over two orders of magnitude, from 0.01 to 1.0. This variation caused the governing dimensionless Biot number β to change from 0.1 to 10, representing a shift from external to internal mass transfer dominance [9,78]. The substrate concentration $S_{0}$ was also independently varied from 0.025 to 3 mM. Simulations were conducted for both types of analysis, injection (IA) and batch (BA), using fixed parameters of $V_{\max}$ = 100μM/s and $K_{I}=K_{M}$. Figure 5 shows the number $N_{\mathrm{ext}}$ of extrema calculated from the simulated responses.

As one can see in Figure 5a, in IA, the dependence of the number $N_{\mathrm{ext}}$ of extrema of the transient current on the Biot number is rather similar in shape to that on the diffusion module $\sigma^{2}$. In IA, when the mass transport by diffusion in the diffusion layer is notably faster than in the enzyme layer (β≳2), the response follows a two-phase pattern ($N_{\mathrm{ext}}=0$). At smaller values of β, when the diffusion in the outer diffusion layer is comparable with or slower than that in the enzyme layer (β≲1), the response follows a three-phase pattern ($N_{\mathrm{ext}}=1$), although it also depends on the substrate concentration. The yellow line in Figure 5a represents an approximate boundary between the two values of $N_{\mathrm{ext}}$, 0 and 1,

(21) $$S_{0}^{*}=3.2+12.7\beta .$$

In the case of BA (Figure 5b), the relationship between the number $N_{\mathrm{ext}}$ of extrema, the Biot number β, and the substrate concentration is noticeably more complex. The number $N_{\mathrm{ext}}$ varies between zero and three, and the boundary between the regions where $N_{\mathrm{ext}}=0$ and $N_{\mathrm{ext}}=1$ is clearly nonlinear. Nevertheless, the boundary (yellow line) between areas indicated as $N_{\mathrm{ext}}=0$ and $N_{\mathrm{ext}}=2$ is similar to that observed in IA (Figure 5a) between $N_{\mathrm{ext}}=0$ and $N_{\mathrm{ext}}=1$. At relatively high substrate concentrations ($S_{0}^{*}≳15$), the number of response phases is the same in both modes of analysis, IA and BA, except when 0.15≲β≲2, where the number of phases in BA is greater than in IA.

**Figure 6:**
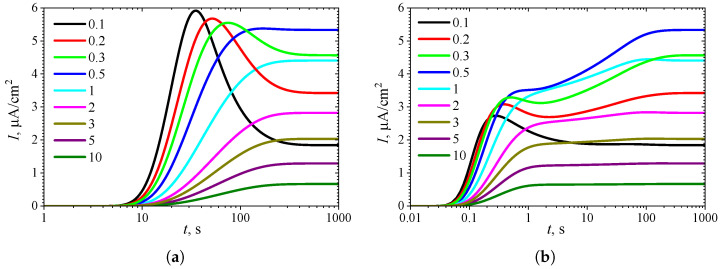
Dynamics of the output current I(t) at nine values of the Biot number β, with fixed substrate concentration $S_{0}^{*}$ = 10 and $K_{I}^{\prime }=\infty$, in IA (**a**) mode and BA (**b**) mode. Other parameters are the same as in Figure 5.

To observe the effect of the Biot number β on the shape of the transient response, the biosensor performance was simulated at nine values of β, while keeping the substrate concentration fixed at a relatively high level ($S_{0}^{*}$ = $10K_{I}^{*}$ = 10). The simulation results are shown in Figure 6.

Figure 6a shows that in IA at $S_{0}^{*}=10$, the transient biosensor current exhibits a global maximum for β≤0.5, whereas it is a monotonously increasing function of time *t* for greater values of the Biot number β, as also shown in Figure 5a.

In BA (Figure 6b), three extrema can be observed only for the smallest value of the Biot number of β=0.1. However, a local minimum and local maximum differ by less than 1 percent. At β=0.5, no local extrema are observed, but an extra inflection point (t≈2 s) is observed where the curve changes from concave down to concave up. When 1≤β≤3, the transient output current has only one global maximum ($N_{\mathrm{ext}}=1$), which occurs noticeably later (at t≈100 s) than the global maximum observed for small values of β (at t<1 s), and is close to the steady-state value. For larger values of β (β>3), the global maximum decreasingly approaches the steady-state value.

In addition to the Biot number β, the external Thiele modulus $\sigma_{\mathrm{ext}}$ (also known as the external Damköhler number and the external diffusion module) is used to compare external and internal mass transport resistances. It relates the characteristic timescale of the enzymatic reaction within the enzyme layer to that of external mass transfer, i.e., it represents the ratio between the first-order surface reaction rate ($V_{\max}\theta_{S}/K_{M}d_{1}$) and the rate of the mass transfer through the external diffusion layer ($D_{S_{2}}/d_{2}$) [22,35,49,50]. If $\sigma_{\mathrm{ext}}^{2}\ll 1$, then the external mass transfer is fast, and the system acts in a reaction-limited regime. The enzymatic reaction is fast, and the external diffusion is limiting when $\sigma_{\mathrm{ext}}^{2}\gg 1$. The internal and external Thiele moduli are related through the Biot number for the mass transfer,

(22) $$\sigma_{\mathrm{ext}}^{2}=\frac{V_{\max}\theta_{S}d_{1}d_{2}}{K_{M}D_{S_{2}}}=\frac{\sigma^{2}}{\beta_{S}}.$$

The effect of the external Thiele modulus on the behavior of amperometric enzyme-based biosensors was not investigated separately, as it is represented through two other dimensionless parameters: the diffusion module $\sigma^{2}$ and the Biot number β = $\beta_{S}$.

### 3.4. Effect of Uncompetitive Substrate Inhibition

**Figure 7:**
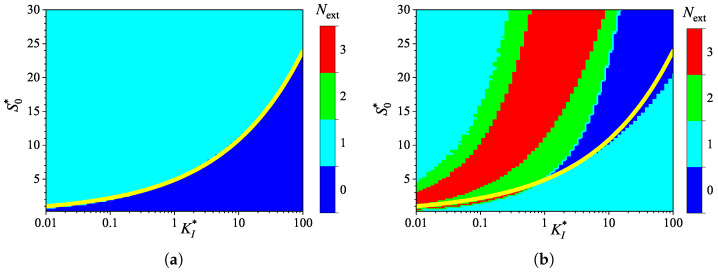
Number $N_{\mathrm{ext}}$ of extrema of the biosensor current vs. the normalized inhibition constant $K_{I}^{*}$ and the normalized substrate concentration $S_{0}^{*}$, with fixed parameters $V_{\max}$ = 100μM/s, θ=0.75 and $K_{I}^{\prime }=\infty$, for IA (**a**) mode and BA (**b**) mode. Other parameters are defined in (19).

To investigate the effect of uncompetitive substrate inhibition ($K_{I}^{\prime }=\infty$) on the behavior of the biosensor transient current, the biosensor response was simulated by varying the inhibition constant $K_{I}$ over four orders of magnitude, from 1μM to 10mM, thereby changing the normalized inhibition constant $K_{I}^{*}$ from 0.01 to 100. The substrate concentration $S_{0}$ was independently varied from 0.025 to 3 mM, as in the numerical experiments discussed above. Simulations were conducted for both types of analysis, injection (IA) and batch (BA), using fixed parameters of $V_{\max}$ = 100μM/s and θ=0.75. At these parameter values, the diffusion module $\sigma^{2}=1$, and the Biot number β=0.13. Figure 7 shows the calculated number $N_{\mathrm{ext}}$ of extrema.

As shown in Figure 7, the dependence of the number $N_{\mathrm{ext}}$ of extrema of the transient current on the inhibition constant $K_{I}^{*}$ differs noticeably from those on the diffusion module $\sigma^{2}$ (Figure 3) and the Biot number β (Figure 5).

In IA (Figure 7a), the response follows a three-phase pattern ($N_{\mathrm{ext}}=1$) in most of the entire region of parameter values, $(S_{0}^{*},K_{I}^{*})\in$[0.25,30]×[0.01,100]. Only at relatively low substrate concentrations and large values of the inhibition constant, the response follows a two-phase pattern ($N_{\mathrm{ext}}=0$), as in the Michaelis–Menten kinetics. This behavior is reasonable because the influence of inhibition decreases with an increasing inhibition constant $K_{I}^{*}$. Nevertheless, an increase in the inhibition constant can be compensated for by an increase in the substrate concentration. In a particular case of $K_{I}^{*}=10$, the inhibitory term $S^{2}/K_{I}$ in the reaction rate Equation (5) becomes significant when $S_{0}^{*}\geq K_{I}^{*}$. The relationship between $S_{0}^{*}$ and $K_{I}^{*}$ is nonlinear. The yellow line in Figure 7a represents an approximate boundary between the two values of $N_{\mathrm{ext}}$, 0 and 1. This line is a power-law (allometric) approximation of the boundary,

(23) $$S_{0}^{*}=4.9\times {K_{I}^{*}}^{0.345}.$$

As shown in Figure 7, in the region parameter values $S_{0}^{*}$ and $K_{I}^{*}$ where $N_{\mathrm{ext}}=0$ in IA (i.e., below the yellow line), $N_{\mathrm{ext}}=1$ in BA.

Figure 7a shows that, in the particular case of $K_{I}^{*}=1$, the transient output current in IA is a monotonically increasing function of time for concentrations $S_{0}^{*}≲5$, and become a non-monotonic function (with $N_{\mathrm{ext}}=1$) at higher concentrations. In the corresponding BA (Figure 7b), the number $N_{\mathrm{ext}}$ changes with increasing substrate concentration in the following sequence: 1, 0, 2, 3. Notably, $N_{\mathrm{ext}}=0$ occurs in the specific case of $S_{0}^{*}=5$. The current dynamics in these cases are also illustrated in Figure 2.

**Figure 8:**
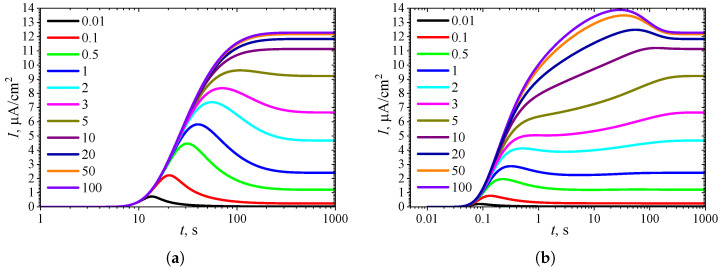
Dynamics of the output current I(t) at eleven values of the normalized inhibition constant $K_{I}^{*}$, with fixed substrate concentration $S_{0}^{*}$ = 10 and $K_{I}^{\prime }=\infty$, in IA (**a**) mode and BA (**b**) mode. Other parameters are the same as in Figure 7.

To observe the effect of the inhibition constant $K_{I}^{*}$ on the dynamics of the transient current, the biosensor action was simulated at eleven different $K_{I}^{*}$ values, with the constant substrate concentration held at $S_{0}^{*}$ = 10. The simulation results are depicted in Figure 8.

As seen in Figure 7a and Figure 8a, in IA at $S_{0}^{*}=10$, the transient current exhibits a global maximum for all values of the inhibition constant $K_{I}^{*}$ less than 8. Only when $K_{I}^{*}$ becomes comparable to or greater than the concentration $S_{0}^{*}$ ($K_{I}^{*}≳S_{0}^{*}$) does the output current become a monotonically increasing function of time *t*.

In BA (Figure 6b and Figure 7b), the transient current at $S_{0}^{*}=10$ is monotonic only within a relatively narrow range of the inhibition constant $K_{I}^{*}$, approximately between 3 and 8. The number of extrema $N_{\mathrm{ext}}=3$ is observed when $K_{I}^{*}$ varies approximately between 0.1 and 0.9. However, the local minimum and maximum differ from the steady-state by only 1–2%, although the global maximum is noticeably more pronounced. Such small deviations in local extrema from the steady value can be considered perturbations of the response, influencing the response analysis procedure [21,35,36,89].

### 3.5. Effect of Noncompetitive Substrate Inhibition

**Figure 9:**
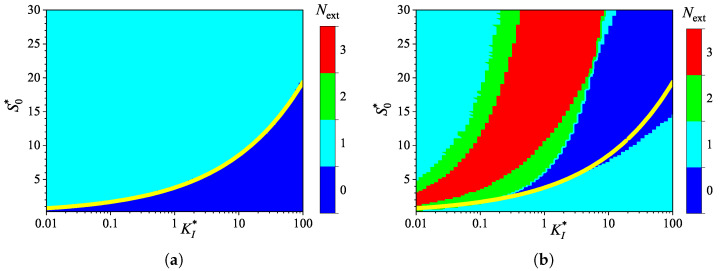
Number $N_{\mathrm{ext}}$ of extrema of the biosensor current vs. the normalized inhibition constant $K_{I}^{*}$ and the normalized substrate concentration $S_{0}^{*}$, with a fixed competitive substrate inhibition constant $K_{I}^{\prime }=K_{M}$ for IA (**a**) mode and BA (**b**) mode. Other parameters are the same as in Figure 7.

**Figure 10:**
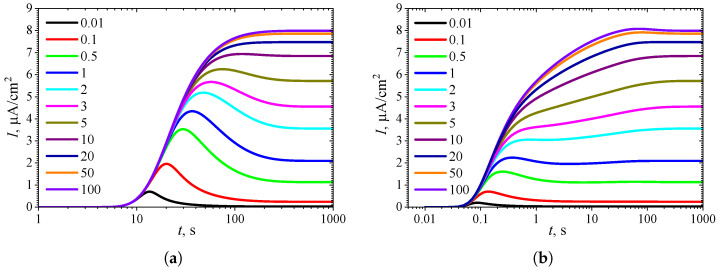
Dynamics of the output current I(t) at eleven values of the normalized inhibition constant $K_{I}^{*}$, with fixed substrate concentration $S_{0}^{*}$ = 10 and $K_{I}^{\prime }=K_{M}$, in IA (**a**) and BA (**b**) modes. Other parameters are the same as in Figure 7 and Figure 9.

**Figure 11:**
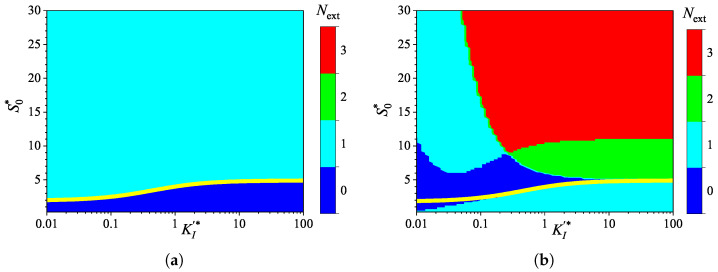
Number $N_{\mathrm{ext}}$ of extrema for the biosensor current vs. the normalized inhibition constant ${K_{I}^{\prime }}^{*}$ and the normalized substrate concentration $S_{0}^{*}$, with a fixed uncompetitive substrate inhibition constant $K_{I}=K_{M}$ for IA (**a**) and BA (**b**) modes. Other parameters are the same as in Figure 9.

**Figure 12:**
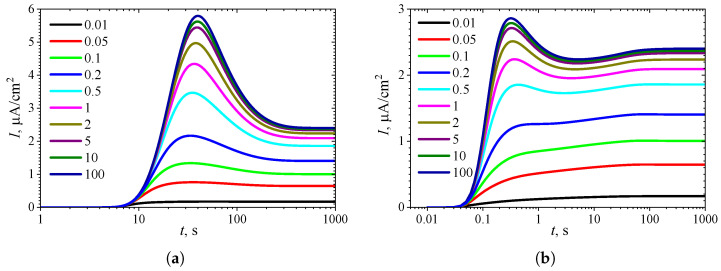
Dynamics of the output current I(t) for ten values of the normalized inhibition constant ${K_{I}^{\prime }}^{*}$, with fixed $S_{0}^{*}$ = 10 and $K_{I}=K_{M}$, in IA (**a**) and BA (**b**) modes. Other parameters are the same as in Figure 11.

To examine the impact of noncompetitive substrate inhibition on the transient current behavior of the biosensor, the response was simulated by varying both the uncompetitive ($K_{I}$ ) and competitive ($K_{I}^{\prime }$) inhibition constants. All other parameters were the same as those used in the analysis of uncompetitive substrate inhibition presented earlier in this section. The simulation results are presented in Figure 9, Figure 10, Figure 11 and Figure 12. Figure 9 and Figure 11 display the computed number $N_{\mathrm{ext}}$ of extrema, while Figure 10 and Figure 12 show the dynamics of the output current I(t).

The numerical experiments whose results are presented in Figure 9 and Figure 10 differ from those shown in Figure 7 and Figure 8 only in the values of the competitive inhibition constant $K_{I}^{\prime }$. Figure 9 and Figure 10 illustrate how the response of the biosensor, operating under uncompetitive substrate inhibition, is influenced by the addition of competitive substrate inhibition at a fixed moderate rate $K_{I}^{\prime }=K_{M}$; that is, how the competitive substrate inhibition reaction (3) affects the response of the biosensor governed by reactions (1) and (2).

One can observe similar shapes in the evolution of output current I(t) in Figure 8 and Figure 10, but there is a noticeable difference in its absolute values. However, this difference becomes significant only when the uncompetitive inhibition constant $K_{I}$ exceeds the Michaelis constant ($K_{I}>K_{M}$, $K_{I}^{*}>1$). At $K_{I}^{*}=0.1$ ($K_{I}=0.1K_{M}$), the steady-state output current $I_{\mathrm{ss}}$ in the case of noncompetitive substrate inhibition (Figure 10) is only about 1% lower than that for uncompetitive substrate inhibition (Figure 8). This difference increases with increasing substrate concentration $S_{0}$, reaching 12% at $K_{I}^{*}=1$ and 35% at $K_{I}^{*}=100$. This property is valid for both IA and BA analysis modes.

The increasing influence of the competitive inhibition constant $K_{I}^{\prime }$ while increasing the uncompetitive inhibition constant $K_{I}$ can also be observed in the dependence of the number $N_{\mathrm{ext}}$ of extrema in the biosensor current on the normalized inhibition constant $K_{I}^{*}$ and the substrate concentration $S_{0}^{*}$ (Figure 7 and Figure 9). The corresponding regions with the same number $N_{\mathrm{ext}}$ have very similar shapes for $K_{I}^{*}<1$, but they diverge for larger values of $K_{I}^{*}$. Similar to the case of uncompetitive inhibition, the boundary between the two values of $N_{\mathrm{ext}}$, 0 and 1, observed in the IA mode for noncompetitive inhibition and indicated by the yellow line in Figure 9a, is also approximated by a power-law (allometric) relationship,

(24) $$S_{0}^{*}=3.81\times {K_{I}^{*}}^{0.354}.$$

The increasing effect of the competitive inhibition constant $K_{I}^{\prime }$ with an increasing uncompetitive inhibition constant $K_{I}$ can be explained by the reaction rate expression (5). At a fixed value of $K_{I}^{\prime }$, the rate of the noncompetitive (mixed) inhition V(S) approaches the rate of competitive inhibition, as defined in (7), when $K_{I}\gg K_{I}^{\prime }$.

To extend the study of the impact of noncompetitive substrate inhibition on the transient current behavior of the biosensor, the response was simulated by varying the competitive ($K_{I}^{\prime }$), keeping the uncompetitive ($K_{I}$ ) inhibition constants unchanged. Figure 11 shows the dependence of the number $N_{\mathrm{ext}}$ of extrema in the biosensor current on the normalized inhibition constant $K_{I}^{*}$ and the normalized substrate concentration $S_{0}^{*}$, with a fixed competitive substrate inhibition constant $K_{I}^{\prime }=K_{M}$ for the IA (**a**) mode and BA (**b**) mode.

As one can see in Figure 11a, in IA, the dependence of the number $N_{\mathrm{ext}}$ of extrema of the transient current on the normalized competitive inhibition constant ${K_{I}^{\prime }}^{*}$ noticeably differs in shape from the dependencies observed earlier in this section. The yellow line in Figure 11a, representing an approximate boundary between the two values of $N_{\mathrm{ext}}$, 0 and 1, saturates as ${K_{I}^{\prime }}^{*}\to \infty$ and is expressed as a rational function,

(25) $$S_{0}^{*}=4.9-\frac{1.3}{0.44+{K_{I}^{\prime }}^{*}}\;.$$

Figure 11b shows a relatively large region where $N_{\mathrm{ext}}=3$ in BA. However, this region corresponds to only relatively high substrate concentrations ($S_{0}^{*}≳10$). At $S_{0}^{*}=10$, Figure 12b clearly shows local minima and maxima for ${K_{I}^{\prime }}^{*}>0.2$ ($N_{\mathrm{ext}}=2$). The difference in maximal currents between the analysis modes is among the most significant differences observed, as shown in (Figure 12), compared to the other cases presented in Figure 2, Figure 4, Figure 6, Figure 8 and Figure 10.

When ${K_{I}^{\prime }}^{*}\gg 1$, at a fixed value of $K_{I}^{*}$, the rate of noncompetitive (mixed) inhibition V(S) approaches the rate of uncompetitive inhibition, as defined in (6). Thus, at ${K_{I}^{\prime }}^{*}=100$, the simulation results shown in Figure 11 and Figure 12 for ${K_{I}^{\prime }}^{*}=100$ coincide with the results presented in Figure 7 and Figure 8 for the specific value $K_{I}^{*}=1$. In particular, the output currents in Figure 12, labeled with 100, coincide with the currents in Figure 8, labeled with 1, as in both cases $K_{I}^{*}=1$ ($K_{I}=K_{M}$).

## 4. Conclusions

The two-compartment mathematical model (8)–(14) of amperometric enzyme-based biosensors is useful for investigating the influence of noncompetitive (mixed) substrate inhibition, in conjunction with internal and external diffusion limitations, on the biosensor response. Deriving the corresponding dimensionless form of the model (A1)–(A7) reveals the main governing dimensionless parameters (18).

The dynamics of the enzymatic system shown in Figure 1 are highly sensitive to internal and external diffusion limitations, enzyme inhibition, and, most notably, the mode of analysis. In injection analysis (IA, real-time monitoring), where the arrival of the analyte initiates the biosensor transient response, the response follows a two- or three-phase pattern. In batch analysis (BA), where the biosensor is directly immersed in a buffer solution containing the analyte, the response may exhibit up to five phases. The number $N_{\mathrm{ext}}$ of extrema in the transient output current, as defined by Equation (17), is useful for determining the conditions under which the biosensor response follows a multi-phase pattern. The steady-state biosensor current is invariant with respect to the analysis mode (Figure 2, Figure 4, Figure 6, Figure 8, Figure 10 and Figure 12).

The non-monotonic (three-phase, $N_{\mathrm{ext}}=1$) transient output current of biosensors operating in IA is observed only when the enzyme kinetics predominate in the biosensor response, compared to the internal diffusion, or when the operation is under mixed control (diffusion module $\sigma^{2}≲10$) (Figure 3a), the diffusion in the outer diffusion layer is comparable to or slower than that in the enzyme layer (the Biot number β≲1) (Figure 5a), and the substrate concentration is comparable to or greater than the inhibition constant (Figure 7a), where the current shows a global maximum greater than a steady value (Figure 4a, Figure 6a and Figure 8a).

In BA, the monotonic (two-phase, $N_{\mathrm{ext}}=0$) transient current is observed only when the biosensor response is notably governed by enzyme kinetics ($\sigma^{2}<1$), mass transport by diffusion in the diffusion layer is faster than in the enzyme layer (β≳1), and under specific values of other parameters. Under conditions where biosensors operating in IA follow a three-phase pattern, the transient current in BA can exhibit a maximum five-phase pattern ($N_{\mathrm{ext}}=3$) (Figure 3b, Figure 5b, and Figure 7b), where the current shows a global maximum, a local minimum, and a local maximum. The global maximum may occur either before or after a local minimum (Figure 4b, Figure 6b, and Figure 8b).

The effect of the competitive substrate inhibition constant $K_{I}^{\prime }$ on the response dynamics of biosensors operating under noncompetitive (mixed) substrate inhibition increases with increasing uncompetitive inhibition constant $K_{I}$ (Figure 9, Figure 10, Figure 11 and Figure 12).

Oscillations in the transient biosensor response, caused by substrate inhibition and both internal and external diffusion limitations, should be taken into consideration when using the biosensor calibration curve.

## Data Availability

The original contributions presented in this study are included in the article. Further inquiries can be directed to the corresponding author.

## Abbreviations

The following abbreviations are used in this manuscript:

E	Enzyme
S	Substrate
P	Reaction product
ES	Enzyme–substrate complex
ESS	Substrate–enzyme–substrate complex
ESI	Substrate-inhibited enzyme complex
IA	Injection analysis
BA	Batch analysis
QSSA	Quasi-steady-state approximation

## Appendix A. Dimensionless Mathematical Model

The two-compartment model (8)–(14) was expressed in the dimensionless form by rescaling the time, space, concentrations, and diffusion coefficients, defined in Table A1.

The governing Equation (8) in the dimensionless form was then expressed as follows ($t^{*}>0$):

(A1) $$\begin{array}{cc} \frac{\partial S_{1}^{*}}{\partial t^{*}} & =\frac{\partial^{2}S_{1}^{*}}{\partial {x^{*}}^{2}}-\sigma^{2}\frac{S_{1}^{*}}{1+S_{1}^{*}/{K_{I}^{\prime }}^{*}+S_{1}^{*}\left(1+S_{1}^{*}/K_{I}^{*}\right)}\;,\\ \frac{\partial P_{1}^{*}}{\partial t^{*}} & =D_{P_{1}}^{*}\frac{\partial^{2}P_{1}^{*}}{\partial {x^{*}}^{2}}+\sigma^{2}\frac{S_{1}^{*}}{1+S_{1}^{*}/{K_{I}^{\prime }}^{*}+S_{1}^{*}\left(1+S_{1}^{*}/K_{I}^{*}\right)}\;,\;x^{*}\in \left(0,\;1\right), \end{array}$$

where $D_{P_{1}}^{*}$ = $D_{P_{2}}/D_{S_{1}}$.

The diffusion Equation (9) takes the following form ($t^{*}>0$):

(A2) $$\begin{array}{cc} \frac{\partial S_{2}^{*}}{\partial t^{*}} & =D_{S_{2}}^{*}\frac{\partial^{2}S_{2}^{*}}{\partial {x^{*}}^{2}},\\ \frac{\partial P_{2}^{*}}{\partial t^{*}} & =D_{P_{2}}^{*}\frac{\partial^{2}P_{2}^{*}}{\partial {x^{*}}^{2}},\;x^{*}\in \left(1,\;a_{2}^{*}\right), \end{array}$$

where $D_{S_{2}}^{*}$ = $D_{S_{2}}/D_{S_{1}}$, $D_{P_{2}}^{*}$ = $D_{P_{2}}/D_{S_{1}}$, $a_{2}^{*}$ = $a_{2}/a_{1}$.

The boundary conditions (10), (11) and (12) are rewritten as follows ($t^{*}>0$):

(A3) $$S_{2}^{*}\left(a_{2}^{*},t^{*}\right)=S_{0}^{*},\;P_{2}^{*}\left(a_{2}^{*},\;t^{*}\right)=0,$$

(A4) $$\begin{array}{cc} \frac{\partial S_{1}^{*}}{\partial x^{*}}{|}_{x^{*}=1} & =D_{S_{2}}^{*}\frac{\partial S_{2}^{*}}{\partial x^{*}}{|}_{x^{*}=1},\;S_{1}^{*}\left(1,t^{*}\right)=\theta_{S}S_{2}^{*}\left(1,t^{*}\right),\\ \frac{\partial P_{1}^{*}}{\partial x^{*}}{|}_{x^{*}=1} & =D_{P_{2}}^{*}\frac{\partial P_{2}^{*}}{\partial x^{*}}{|}_{x^{*}=1},\;P_{1}^{*}\left(1,t^{*}\right)=\theta_{P}P_{2}^{*}\left(1,t^{*}\right), \end{array}$$

(A5) $$P_{1}^{*}\left(0,t^{*}\right)=0,\;\frac{\partial S_{1}^{*}}{\partial x^{*}}{|}_{x^{*}=0}=0.$$

The initial conditions specific to IA, as given in (13), take the following form:

(A6) $$\begin{array}{cc} \; & S_{1}^{*}\left(x^{*},0\right)=0,\;\;\;P_{1}^{*}\left(x^{*},0\right)=0,\;x^{*}\in \left[0,\;1\right],\\ \; & S_{2}^{*}\left(x^{*},0\right)=0,\;\;\;P_{2}^{*}\left(x^{*},0\right)=0,\;x^{*}\in \left[1,a_{2}^{*}\right),\\ \; & S_{2}^{*}\left(a_{2}^{*},\;0\right)=S_{0}^{*},\;P_{2}^{*}\left(a_{2}^{*},\;0\right)=0. \end{array}$$

The initial conditions (14), which are specific to BA, are transformed to the following conditions:

(A7) $$\begin{array}{cc} \; & S_{1}^{*}\left(x^{*},0\right)=0,\;\;P_{1}^{*}\left(x,0\right)=0,\;x^{*}\in \left[0,\;1\right),\\ \; & S_{1}^{*}\left(1,\;0\right)=\theta_{S}S_{0}^{*},\;P_{1}^{*}\left(1,\;0\right)=0,\\ \; & S_{2}^{*}\left(x^{*},0\right)=S_{0}^{*},\;\;\;P_{2}^{*}\left(x^{*},0\right)=0,\;x^{*}\in \left[1,a_{2}^{*}\right]. \end{array}$$

**Table A1:** Dimensional and dimensionless parameters.

Parameter	Dimensional	Dimensionless
Time	*t*, s	$t^{*}=D_{S_{1}}t/a_{1}^{2}$
Distance from electrode	x,μm	$x^{*}=x/a_{1}$
Enzyme layer thickness	$d_{1},\;\mu m$	$d_{1}^{*}=d_{1}/a_{1}=1$
Diffusion layer thickness	$d_{2},\;\mu m$	$d_{2}^{*}=d_{2}/a_{1}$
Substrate concentration in enzyme layer	$S_{1},\;\mu M$	$S_{1}^{*}=S_{1}/K_{M}$
Product concentration in enzyme layer	$P_{1},\;\mu M$	$P_{1}^{*}=P_{1}/K_{M}$
Substrate concentration in diffusion layer	$S_{2},\;\mu M$	$S_{2}^{*}=S_{2}/K_{M}$
Product concentration in diffusion layer	$P_{2},\;\mu M$	$P_{2}^{*}=P_{2}/K_{M}$
Substrate concentration in bulk	$S_{0},\;\mu M$	$S_{0}^{*}=S_{0}/K_{M}$
Michaelis constant	$K_{M},\;\mu M$	$K_{M}^{*}=K_{M}/K_{M}=1$
Uncompetitive substrate inhibition constant	$K_{I},\;\mu M$	$K_{I}^{*}=K_{I}/K_{M}$
Competitive substrate inhibition constant	$K_{I}^{\prime },\;\mu M$	${K_{I}^{\prime }}^{*}=K_{I}^{\prime }/K_{M}$
Maximal enzymatic rate	$V_{\max},\;\mu M/s$	
Current density	$I,\;\mu A/cm^{2}$	$I^{*}=Id_{1}/\left(n_{e}FD_{S_{1}}K_{M}\right)$
Steady-state current density	$I_{\mathrm{ss}},\;\mu A/cm^{2}$	$I_{\mathrm{ss}}^{*}=I_{\mathrm{ss}}d_{1}/\left(n_{e}FD_{S_{1}}K_{M}\right)$
Diffusion coefficient of substrate in enzyme layer	$D_{S_{1}},\;\mu m^{2}/s$	$D_{S_{1}}^{*}=D_{S_{1}}/D_{S_{1}}=1$
Diffusion coefficient of product in enzyme layer	$D_{P_{1}},\;\mu m^{2}/s$	$D_{P_{1}}^{*}=D_{P_{1}}/D_{S_{1}}$
Diffusion coefficient of substrate in diffusion layer	$D_{S_{2}},\;\mu m^{2}/s$	$D_{S_{2}}^{*}=D_{S_{2}}/D_{S_{1}}$
Diffusion coefficient of product in diffusion layer	$D_{P_{2}},\;\mu m^{2}/s$	$D_{P_{2}}^{*}=D_{P_{2}}/D_{S_{1}}$
Partition coefficient for substrate		$\theta_{S}$
Partition coefficient for product		$\theta_{P}$
Biot number for substrate		$\beta_{S}=D_{S_{2}}d_{1}/\left(\theta_{S}D_{S_{1}}d_{2}\right)$
Biot number for product		$\beta_{P}=D_{P_{2}}d_{1}/\left(\theta_{P}D_{P_{1}}d_{2}\right)$
Diffusion module		$\sigma^{2}=V_{\max}d_{1}^{2}/\left(K_{M}D_{S_{1}}\right)$
External diffusion module		$\sigma_{\mathrm{ext}}^{2}=\sigma^{2}/\beta_{S}$
